# Multidisciplinary prehabilitation to improve frailty and functional capacity in high-risk elective surgical patients: a retrospective pilot study

**DOI:** 10.1186/s13741-024-00359-x

**Published:** 2024-01-23

**Authors:** Henry Man Kin Wong, Ding Qi, Bosco Hon Ming Ma, Pik Yi Hou, Calvin Ka Woon Kwong, Anna Lee, Prehab Study Group, Stefanie So Ling Lam, Terry Ho Yan Ting, Kenny Wing Moon Ip, Suet Yi Chan, Peggy Pui Kee Tsung, Albert Kam Ming Chan, Vivian Nga Man Lau, Maria Wing Sze Tang, Kelvin Kwok Chai Ng, Hon Chi Yip, Chi Hang Yee, Gavin Matthew Joynt

**Affiliations:** 1 Department of Anaesthesia and Intensive Care, Prince of Wales Hospital, New Territories, Hong Kong; 2 Department of Anaesthesia and Intensive Care, The Chinese University of Hong Kong, New Territories, Hong Kong; 3 Department of Medicine and Geriatrics, Shatin Hospital, New Territories, Hong Kong; 4 Department of Physiotherapy, Shatin Hospital, New Territories, Hong Kong; 5 Department of Dietitian, Shatin Hospital, New Territories, Hong Kong; 6 Department of Occupational Therapy, Shatin Hospital, New Territories, Hong Kong; 7 Department of Surgery, The Chinese University of Hong Kong, New Territories, Hong Kong; 8 General Surgery, CUHK Medical Centre, New Territories, Hong Kong

**Keywords:** Preoperative exercise, Frailty, Perioperative medicine

## Abstract

**Background:**

Frailty is associated with worse outcomes and higher healthcare costs. The long waiting time for surgery is a potential ‘teachable’ moment. We examined the feasibility and safety of a pilot prehabilitation programme on high-risk frail patients undergoing major elective surgery.

**Methods:**

A single-centre, retrospective pilot study (Dec 2020–Nov 2021) on a one-stop prehabilitation programme (structured exercise training, nutritional counselling/therapy, and psychological support) in collaboration with geriatricians and allied health professionals. At least 4 weeks before surgery, patients at high risk of frailty or malnutrition, or undergoing major hepatectomy, esophagectomy, pancreaticoduodenectomy, or radical cystectomy, were referred for prehabilitation (2–3 sessions/week). The primary outcomes were the feasibility and safety of prehabilitation. The secondary outcomes were changes in functional, emotional, and nutritional status and days alive and at home within 30 days after surgery (DAH_30_) associated with prehabilitation.

**Results:**

Over a 12-month period, 72 out of 111 patients (64.9%) from the Perioperative Medicine Clinic were eligible for prehabilitation, of which 54 (75%) were recruited. The mean (standard deviation) age was 71.9 (6.9) years. The adherence rate to 3 weeks of prehabilitation was high in 52 (96.3%) participants. Prehabilitation improved exercise capacity (*P* = 0.08), enhanced some functional mobility measures (*P* = 0.02), and increased nutritional energy (*P* = 0.04) and protein intakes (*P* < 0.01). However, prehabilitation-related changes in muscle strength, cognitive function, and emotional resilience were minimal. The median (interquatile range) DAH_30_ was 19 (14–23) days. No adverse events were reported.

**Conclusions:**

This outpatient-based, one-stop multidisciplinary prehabilitation programme was feasible, safe, and improved several measures of patient’s physiological reserve and functional capacity.

**Clinical trial registration:**

NCT05668221.

## Background

With an ageing population (Wong K. et al. 2019), there is an increased risk of frailty and a loss in functional and physiological reserve and adaptability (Rockwood et al. 2005). This puts a patient who is exposed to a stressor, such as a major operation, at higher risk of adverse outcomes (Rockwood. et al. 2005). Frailty is associated with a two- to sixfold increased risk of major adverse cardiac and cerebrovascular events, longer hospital and intensive care unit stay, and higher in-hospital and 1-year mortality (Sepehri A. et al. 2014; Rodrigues et al. 2017).

The long surgical waiting time creates a potential ‘teachable moment’ to address problems of low physical fitness, poor nutritional status, and high emotional distress (Levy et al. 2021). High-risk patients are more likely to be identified and optimized in the preoperative period after re-engineering a perioperative pathway and establishing a common platform for multidisciplinary team collaboration (Lee et al. 2011).

Prehabilitation is a new multidisciplinary approach in Hong Kong involving anaesthesiologists, physicians, surgeons, nurses, physiotherapists, occupational therapists, and dietitians. It encompasses multimodal features. First, individualized aerobic and resistance training can enhance cardiopulmonary fitness so that patients can better withstand the stress of surgery (Yau et al. 2021) to lower the risk and severity of postoperative complications (Barberan-Garcia et al. 2018; Molenaar et al. 2022). Second, dietary interventions can help prepare and optimize the patients’ nutritional status to reduce the risk of adverse outcomes due to a high catabolic state and systemic inflammatory response from surgery (Gillis et al. 2022). Malnutrition is associated with postoperative complications after pneumonectomy and hepatectomy (Bagan et al. 2013; Fukami et al. 2021), longer length of stay, higher risk of readmissions, and a higher risk of mortality up to 90 days after surgery (Ting et al. 2019; Ho et al. 2015; Leandro-Merhi and Aquino 2014). Finally, psychological support includes the provision of emotional support to improve the patient’s resilience and advice on behavioural changes such as cessation of smoking and alcohol abuse (Yau et al. 2021, Barberan-Garcia et al. 2018; Molenaar et al. 2023).

Changes in perioperative management require buy-in from hospital administrators and all stakeholders. Therefore, we conducted a pilot study to evaluate the feasibility and safety of a prehabilitation programme on our patients before major elective surgery. The purpose was to provide an example service model for clinicians’ consideration for the wider uptake of such programmes in the public hospitals’ perioperative systems. The primary objectives were to estimate the recruitment, attrition and adherence rates, and safety of prehabilitation. The secondary objectives were to describe the changes in functional, emotional, and nutritional status and days alive and at home within 30 days after surgery (DAH_30_) associated with prehabilitation (Myles et al. 2017).

### Methods

After obtaining approval for the study from the Joint Chinese University of Hong Kong-New Territories East Cluster Research Ethics Committee (CREC Ref. No. 2021.493), the records of patients attending the Perioperative Medicine Clinic (POMC) from December 2020 to November 2021 were reviewed. This was a single-centred, unblinded, retrospective pilot study conducted at the Prince of Wales Hospital (PWH), a university teaching hospital in Hong Kong. In the routine preoperative care pathway, surgical referrals were managed by anaesthetists in the preoperative assessment clinic. Whenever medical optimization was necessary, physicians were consulted for assessments. Allied health professional referrals were initiated by surgeons at the time a decision for surgery was made. Patients at risk of preoperative malnutrition were not routinely screened. No preoperative interventions were prescribed to frail patients with poor functional capacity.

### Perioperative medicine clinic (POMC)

**Fig. 1:**
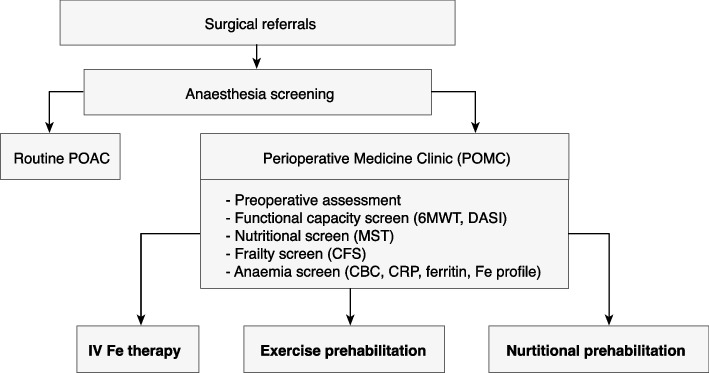
Workflow of prehabilitation programme. POAC, preoperative assessment clinic; 6MWT, 6-min walk test; DASI, Duke Activity Status Index; MST, Malnutrition Screening Tool; CFS, Clinical Frailty Scale; CBC, complete blood count; CRP, C-reactive protein

The new preoperative care pathway has been piloted since November 2020 (Fig. 1). Patients were screened by anaesthesiologists for inclusion into the POMC at the PWH at least 4 weeks before surgery. At the POMC, patients received malnutrition screening using the Malnutrition Screening Tool (MST) (Ferguson et al. 1999), frailty screening with the Clinical Frailty Scale (CFS) (Juma et al. 2016; Rockwood et al. 2005), functional capacity screening using the 6-min walk test (6MWT) (Bohannon and Crouch 2017), and Duke Activity Status Index (DASI) (Wijeysundera et al. 2020). CFS is a measure of fitness and frailty in elderly people. It is a 7-point scale that can be quickly used to assess frailty validly and reliably (Aucoin et al. 2020). The 6MWT is a submaximal exercise test used to assess aerobic capacity and endurance. The distance covered over a time of 6 min has been widely used to estimate exercise capacity which correlates with the results of cardiopulmonary exercise testing (Ross et al. 2010). DASI is a self-reported questionnaire that measures a patient’s functional capacity. A cutoff of 34 is a threshold for identifying patients at risk of postoperative cardiovascular complications and death (Wijeysundera et al. 2020). All patients were required to have an estimated 5 weeks or more surgical waiting time to ensure an optimal prehabilitation time of 3 weeks or more.

### Prehabilitation inclusion and exclusion criteria

The inclusion was based on the type of scheduled surgical procedure and the patient’s functional health status. Procedure-specific inclusions included major hepatectomy (resection of three or more Couimaud’s segments), pancreaticoduodenectomy, esophagectomy, and radical cystectomy. We also included adult patients aged 50 or older, undergoing elective major procedures from the following surgical subspecialties (hepatobiliary, upper gastrointestinal, colorectal, and urological), together with one of the following:

1. American Society of Anaesthesiologists physical status (ASA-PS) score ≥ 3
2. Pre-frail to moderately frail patients (Clinical Frailty Scale 3–6) at the time of assessment at the POMC
3. 6MWT < 400 m
4. DASI < 34 (maximum score = 58.2)
5. MST ≥ 2 (maximum score = 5)

The exclusion criteria were as follows:

1. Unstable angina or unstable cardiac syndrome (New York Heart Association classification IV), critical left main coronary disease, hospitalization for arrhythmias, congestive heart failure, or acute coronary syndrome)
2. Left ventricular outflow obstruction (severe aortic stenosis, hypertrophic cardiomyopathy)
3. Chronic obstructive pulmonary disease Global Initiative for Chronic Obstructive Lung Disease (GOLD) classification stage IV
4. Abdominal aortic aneurysm > 8 cm or suspected dissecting or leaking aortic aneurysm
5. Cognitive deficits unable to comply with study procedures, physical limitations that would preclude prehabilitation, and inability to regularly attend prehabilitation sessions
6. Poor renal function, poor glycaemic control, and severely impaired liver function were excluded from nutritional prehabilitation.

### Exercise prehabilitation

We collaborated with geriatricians at the Geriatric Day Hospital (GDH) at Shatin Hospital for prehabilitation. GDH provides a one-stop comprehensive assessment by geriatricians, physiotherapists, occupational therapists, and dietitians. Eligible patients received extra care at GDH, approximately 3.4 km from PWH, within 1 week after POMC attendance. Patients underwent structured preoperative exercise training of 2 or more weeks, depending on the surgical schedule, to optimize their physical and psychosocial fitness. The prehabilitation was individualized and symptom limited, in which the exercise prescription and progression were based on results of the 6MWT to estimate the patient’s peak oxygen uptake (VO_2peak_) and hence oxygen uptake reserve (VO_2_R), individual health status, exercise performance, and training response. Based on the American College of Sports Medicine guidelines (American College of Sports Medicine 2018; Wong et al. 2019), the exercise protocol comprised of 75 to 90 min of supervised exercises at least twice per week (three times per week for the first 2 weeks). There was a combination of moderate-intensity aerobic exercise and resistance training, with an exercise intensity of three to six on the modified Borg Scale. For aerobic exercise, the patients were asked to perform treadmill walking, stepping exercises, and ergometer for 30 min. For resistance training, patients were asked to perform 10 repetitions per major muscle group of upper and lower limbs with at least 2 sets, depending on individual tolerance and performance. Patients were prescribed a home-based exercise programme that included stretching, aerobic and resistance training, and breathing exercises with guided videos. They were also advised on smoking cessation and bestowed with positive psychology support.

### Psychological prehabilitation

Patients who participated in the prehabilitation programme were given information about the objectives of prehabilitation and the expected physical and nutritional interventions that they would go through during the attendance at the POMC. It was believed that the patients would be better prepared psychologically for surgery if they understood the rationale for preoperative optimization. Patients might be less anxious about the operation if they better understood their risks, and that the risks could be modified and optimized.

### Nutritional prehabilitation

Patients with a MST ≥ 2 were referred to the GDH dietitians for nutritional prehabilitation. The first session was scheduled 4 weeks before the surgery and the second session 1 week before surgery. The dietician’s assessments included nutritional status, anthropometric measurement, body composition analysis, and dietary intake. At the first and second sessions, the target energy level of 25 to 30 kcal/kg/body weight and protein level of 1 to 1.5 g/kg/body weight were prescribed. At the second session, immunonutrition support containing arginine, nucleotides, and omega-3 fish oil was prescribed to metabolically prepare the patient for the surgical stress.

#### Primary outcomes

The primary outcomes were the feasibility and safety of the prehabilitation programme by examining the recruitment, attrition, and adherence of patients to prehabilitation sessions. The reasons for any premature termination of prehabilitation were recorded. Table 1 shows an overview of the assessments performed.

**Table 1:** Assessments overview

**Assessment**	**Baseline**	**First prehab session**	**Last prehab session**	**Clinical outcomes**
**Enrolment**				
Eligibility screen	X			
Demographic data	X			
Comorbidities data	X			
**Primary outcomes**				
Feasibility measures (recruitment rate, attrition rate, compliance rate, reasons for not participating, adverse events)	X	X	X	
**Secondary outcomes**				
6MWT	X	X	X	
DASI	X			
CFS	X			
Other frailty measures (hand grip strength, 30-s chair stand test, time-up-and-go test)		X	X	
DASS-21		X	X	
Nutritional status (skeletal muscle mass, body fat mass, BMI, weight)		X	X	
3-day food record		X	X	
Length of hospital stay				X
30-day mortality				X
DAH_30_				X

*Abbreviations*: *6MWT* 6-min walk test, *DASI* Duke Activity Status Index, *CFS* Clinical Frailty Scale, *DASS* Depression Anxiety Stress Scale, *BMI* body mass index, *DAH*_*30*_ number of days alive and at home within 30 days after surgery

#### Secondary outcomes

##### Functional status

We assessed the change in 6MWT before and after prehabilitation to measure the ability of an individual to maintain a moderate level of physical endurance (ATS statement guidelines 2016). Participants were asked to walk 30 m back and forth in a hallway for 6 min at a pace that made them tired by the end of the walk. A change in 6MWT of 14 m was considered to be clinically meaningful (Bohannon and Crouch 2017). Changes in muscle strength (hand grip strength and 30-s chair stand tests) and changes in functional mobility measures, such as the Timed Up and Go (TUG) (Bohannon 2006), Modified Functional Ambulatory Category (mFAC) (Chau et al. 2013), modified Rivermead Mobility Index (mRMI) (Lennon and Johnson 2000), Morse Fall Scale (MFS) (Jewell et al. 2020), Modified Barthel Index for activity daily living (MBI) (Ohura et al. 2017), and the Lawton Instrumental Activities of Daily Living (Law A. D. L.) (Graf 2008) scores were also recorded.

##### Emotional resilience

Emotional resilience was measured with the Chinese (Hong Kong) version of the Depression Anxiety Stress Scale (DASS-21) before and after prehabilitation as an indicator of psychological stress (Lovibond and Lovibond 1995). This self-reported questionnaire has three subscales corresponding to depression, anxiety, and stress. Each of the three subscales has seven items. Patients were asked to rate each item on a 4-point scale (0 to 3) based on their experience during the past week. The scores on DASS-21 were multiplied by 2 to calculate the final subscale scores, producing a maximum of 42 points in each subscale. The higher the score, the greater the severity of depression, anxiety, and stress.

##### Nutritional status

Nutritional status before and after prehabilitation was measured using body weight, body mass index (BMI), skeletal muscle mass (SMM), and percentage of body fat (%). A dietary assessment was also performed using a 3-day food record. Patients were asked to record all foods and beverages consumed over 3 days before and after prehabilitation, from which the energy and protein intake were estimated.

##### Clinical outcomes

The number of patients that needed preoperative medical optimization, postoperative length of stay, 30-day mortality, and the DAH_30_ was recorded using the data from the Clinical Management System. DAH_30_ is a patient-centred composite measure incorporating length of stay in the hospital following index surgery; readmission to either the index or other hospital, including post-acute hospital discharge to a rehabilitation centre; and early deaths after surgery, into a single outcome metric (Myles et al. 2017).

#### Sample size and statistical analysis

Using PASS 2019 software (NCSS, LLC, Kaysville, UT, USA), a sample size of 44 achieves 90% power to detect a mean of paired differences of 25.2 with an estimated standard deviation of paired differences of 50.2 (medium effect size of 0.50) from a previous prehabilitation study (Gillis et al. 2014) and with a significance level of 0.05 using a two-sided paired *t*-test. Assuming a 20% drop-out rate, we increased the sample size to 55 patients. Categorical data were reported as numbers and percentages. Continuous variables were reported as mean (standard deviation) or median (interquartile range) as appropriate after checking for normality using the Shapiro–Wilk’s test. Generalized estimating equations (GEE) with a Gaussian distribution, identity-link function, exchangeable correlation, and robust variance were used to assess the pre-post changes in physical prehabilitation-related outcome measures after adjusting for CFS (centred at the median of 3) and the number of prehabilitation sessions attended. To avoid overfitting GEE models from a smaller sample of patients referred to nutritional prehabilitation, we adjusted the results only for CFS (centred at the median of 3). Data were analysed using SPSS 27.0 software (IBM Corp, Armonk, NY, USA) and Stata 17.0 (StataCorp, College Station, TX, USA). Although the level of significance was set at *P* < 0.05, we interpreted borderline significance using the terminology outlined by Pocock and Ware (Pocock and Ware 2009).

## Results

**Fig. 2:**
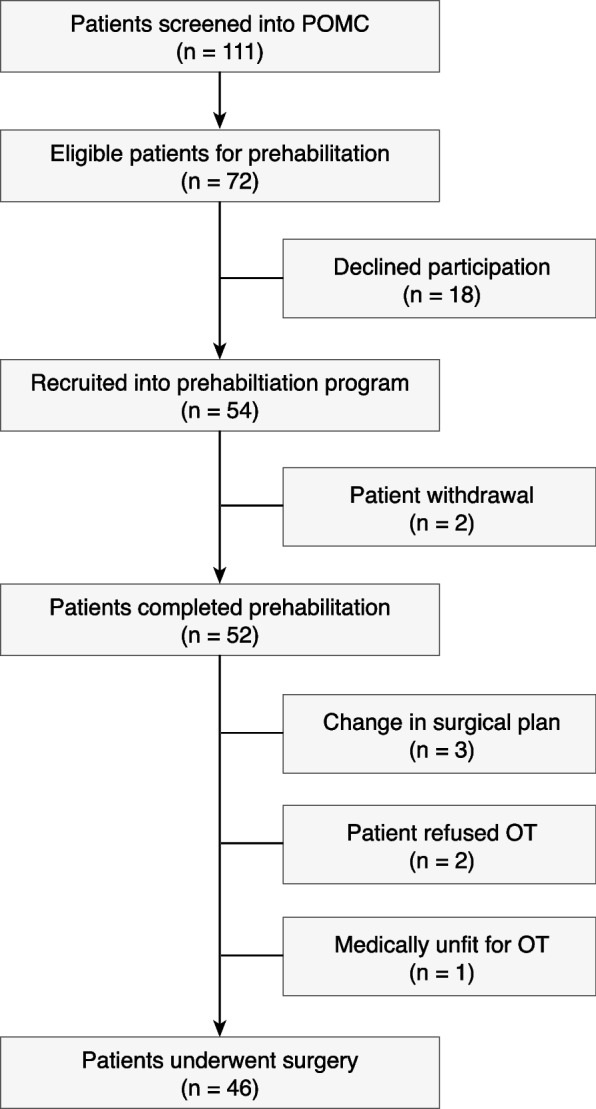
Flow of participants from screening until surgery. POMC, perioperative medicine clinic; OT, operation

Over 12 months, 111 patients were seen in POMC. Seventy-two patients were identified as eligible for prehabilitation, of which 54 (75%) underwent prehabilitation and were retrospectively analysed in this feasibility trial (Fig. 2). The reasons for not participating in prehabilitation in 18 patients were as follows: difficult transport and geographical reasons (*n* = 4), preference of undergoing the surgery in private hospitals (*n* = 2), inconvenience from medical disabilities such as faecal incontinence and renal dialysis (*n* = 2), change in surgical plan (*n* = 1) and new surgical findings (*n* = 1), subjective feeling of being ‘too fit for prehabilitation’ (*n* = 2), and unwillingness to participate (*n* = 6). Of the 54 participants, 52 (96.3%) completed the prehabilitation. Many participants (53.8%) did not finish the intended number of sessions due to the advancement of surgery. The mean (SD) duration of prehabilitation and the number of prehabilitation sessions attended per participant were 20.3 (11.1) days and 6.3 (2.9), respectively. Thirteen participants attended nine sessions or more. Of the 31 (57%) participants referred for nutritional prehabilitation, the median (IQR) duration of nutritional prehabilitation was 14 (14–14) days; 18 (58.1%) participants required immunonutritional support. No adverse events related to prehabilitation were reported.

The demographics and clinical characteristics of the participants, who completed prehabilitation, are presented in Tables 2 and 3. Sixteen (31%) out of 52 participants were pre-frail to frail (*CFS* ≥ 4). The median (IQR) CFS was 3 (3 to 4). Sixteen (31%) participants needed a medical review before surgery. Participants were managed by geriatricians at the same prehabilitation sessions in GDH. Eleven participants required medication titration with or without investigations for their comorbidities (diabetes, hypertension, and asthma), including bradycardia (1), urinary tract infection (1), fluid overload (1), and atrial fibrillation (1).

**Table 2:** Characteristics of patients completed a prehabilitation program

	52 participants
Age (years), mean (SD)	71.9 (6.8)
Gender male, *n* (%)	34 (65.4)
Weight (kg), mean (SD)	62.7 (12.1)
Height (cm), mean (SD)	161.1 (8.0)
BMI (kg m^−2^), mean (SD)	24.1 (4.1)
Smoking status, *n* (%)	
Non-smoker	29 (55.8)
Ex-smoker > 1 year	15 (28.8)
Active smoker	8 (15.4)
Comorbidities, *n* (%)	
Hypertension	35 (67.3)
Diabetes mellitus	29 (55.8)
Asthma/COPD	4 (7.7)
Ischaemic heart disease	6 (11.5)
Atrial fibrillation	6 (11.5)
Cerebrovascular disease	3 (5.8)
Renal impairment	8 (15.4)
Clinical Frailty Scale, *n* (%)	
CFS 2	4 (7.7)
CFS 3	32 (61.5)
CFS 4	13 (25.0)
CFS 5	3 (5.8)
ASA-PS, *n* (%)	
ASA I	1 (1.9)
ASA II	27 (51.9)
ASA III	23 (44.2)
ASA IV	1 (1.9)
Type of surgery, *n* (%)	
Hepatobiliary	27 (51.9)
Upper gastrointestinal	8 (15.4)
Colorectal	13 (15.0)
Urology	4 (7.7)

*Abbreviations:* *SD *Standard deviation, *ASA-PS *American Society of Anaesthesiologists Physical Status, *BMI *Body mass index, *COPD* Chronic obstructive pulmonary disease

**Table 3:** Changes in outcome measure associated with prehabilitation^a^

	Baseline (95% *CI*)	End of prehabilitation (95% *CI*)	Difference (95% *CI*)	*p*-value
**Exercise capacity**				
6MWT (m) (*n* = 52)	393.6 (362.8 to 424.4)	429.3 (399.4 to 459.2)	35.7 (-4.6 to 75.9)	0.082
VO_2peak_ (ml kg^−1^ min^−1^) (*n* = 52)	10.9 (10.0 to 11.8)	11.1 (10.2 to 12.0)	0.2 (-0.8 to 1.2)	0.686
**Muscle strength**				
Right hand grip strength (kg) (*n* = 52)	23.7 (21.3 to 26.2)	25.6 (22.9 to 28.3)	1.9 (-0.9 to 4.6)	0.180
Left hand grip strength (kg) (*n* = 52)	22.9 (20.4 to 25.4)	25.2 (22.5 to 27.9)	2.3 (-0.4 to 5.0)	0.094
30-s chair stand (times) (*n* = 52)	12.0 (10.8 to 13.3)	13.2 (11.7 to 14.6)	1.1 (-0.8 to 3.0)	0.245
**Functional mobility**				
Timed Up and Go (s) (*n* = 52)	10.8 (9.5 to 12.0)	8.7 (7.5 to 9.9)	-2.1 (-3.7 to-0.4)	0.015
mFAC (*n* = 52)	6.6 (6.4 to 6.8)	6.9 (6.6 to 7.1)	0.3 (-0.1 to 0.7)	0.195
mRMI (*n* = 52)	38.6 (38.1 to 39.2)	39.4 (39.0 to 39.9)	0.8 (0.1 to 1.5)	0.022
MFS (*n* = 51)	15.8 (12.7 to 18.9)	14.6 (11.1 to 18.2)	-1.1 (-3.2 to 0.9)	0.264
mBI (*n* = 51)	98.7 (97.5 to 99.8)	99.6 (98.5 to 100.8)	1.0 (-0.9 to 2.8)	0.313
LawADL (*n* = 51)	6.0 (5.6 to 6.4)	6.2 (5.8 to 6.5)	0.2 (0.0 to 0.4)	0.080
**Cognitive function**				
MOCA (*n* = 52)	21.8 (20.7 to 22.9)	22.5 (21.2 to 23.8)	0.7 (-0.7 to 2.2)	0.303
**Emotional resilience**				
DASS_Depression (*n* = 50)	6.4 (3.6 to 9.1)	4.8 (2.3 to 7.4)	-1.5 (-5.5 to 2.5)	0.461
DASS_Anxiety (*n* = 50)	6.4 (4.2 to 8.5)	5.7 (3.2 to 8.2)	-0.7 (-4.3 to 2.9)	0.710
DASS_Stress (*n* = 50)	6.4 (3.3 to 9.5)	7.7 (4.9 to 10.6)	1.3 (-3.6 to 6.3)	0.598
**Nutritional status outcomes**				
Weight (kg)	59.8 (56.1 to 63.5)	59.4 (55.8 to 63.0)	-0.4 (-0.9 to 0.2)	0.193
BMI (kg.m^−2^) (*n* = 31)	23.1 (21.9 to 24.3)	22.7 (21.6 to 23.9)	-0.4 (-0.6 t-0.1)	0.018
Skeletal muscle mass (kg) (*n* = 28)	18.5 (17.0 to 20.0)	18.6 (16.4 to 20.9)	0.1 (-2.1 to 2.3)	0.899
Fat percentage of weight (%) (*n* = 28)	24.5 (20.7 to 28.3)	24.0 (20.2 to 27.8)	-0.5 (-1.9 to 0.8)	0.450
Daily energy intake (kcal.body weight^−1^) (*n* = 31)	26.9 (25.2 to 28.5)	28.3 (26.5 to 30.1)	1.4 (0.1 to 2.8)	0.038
Daily protein intake (g.body weight^−1^) (*n* = 31)	1.1 (1.0 to 1.2)	1.2 (1.1 to 1.2)	0.1 (0 to 0.1)	0.006

*Abbreviations*: *mFAC* Modified Functional Ambulatory Category, *mRMI* Modified Rivermead Mobility Index, *MFS* Morse Fall Scale, *MBI* Modified Barthel Index for activity daily living, *LawADL* Lawton Instrumental Activities of Daily Living, *MOCA* Montreal Cognitive Assessment

^a^Adjusted results using generalized estimating equations

Prehabilitation-related changes in exercise capacity, muscle strength, functional mobility and ambulatory abilities, emotional resilience, cognition, and nutrition status are shown in Table 3. The median (IQR) duration of postoperative length of stay and DAH_30_ were 11 (7–16) and 19 (14–23) days, respectively. One patient died within 30 days after surgery.

## Discussion

The first outpatient-based multidisciplinary prehabilitation programme in Hong Kong shows that it has good feasibility and safety for patients undergoing noncardiac surgery. Participants completed about six prehabilitation sessions over 3 weeks before surgery. The high compliance rate (> 95%) to outpatient exercise training protocol and the overall low drop-out rate (< 5%) suggest that there was high patient acceptability for prehabilitation. Approximately, one-third of participants required medical optimization by the geriatricians. Functional mobility and nutritional status were positively associated with prehabilitation, while some improvements were observed in exercise capacity. Meanwhile, there were minimal changes in muscle strength, cognitive function, and emotional resilience.

Most of the eligible prehabilitation patients (72%) completed the intervention successfully, a similar proportion found in other pilot studies for outpatient and home-based exercise training (Argudo et al. 2020; Chmelo et al. 2022) but lower than the figures reported in the prehabilitation arm of several randomized controlled trials (RCTs) (Barberan-Garcia et al. 2018; Gillis et al. 2014; Kim et al. 2009; Molenaar et al. 2023). The referral rate for nutritional prehabilitation in the study (57%) was comparable to that of a recent Danish prospective controlled study of multimodal prehabilitation (49%) for colorectal cancer patients (Bojesen et al. 2022).

This one-stop outpatient prehabilitation programme was designed to minimize patients’ travel and waiting times for multidisciplinary consultations in contrast to our old service model where there was up to a 2-week interval between anaesthesia preoperative assessments and medical specialty consultations. Some patients did not participate in prehabilitation because it involved travelling multiple times to GDH, a consistent barrier found in other studies (Chmelo et al. 2022; Olsen et al. 2023). Other reasons for nonparticipation, such as waiting time, lack of motivation, illness, and change in surgery plans, were difficulties which were also highlighted in a recent scoping review of cardiac surgery prehabilitation studies (Olsen et al. 2023). The use of home-based physical prehabilitation and telemedicine consultations, improving coordination between surgeons and physiotherapists in scheduling the date of operation and financial support for multiple outpatient visits, is potential strategies to increase the patients’ participation and acceptability for prehabilitation (Olsen et al. 2023; Yau et al. 2019).

Our physical prehabilitation may be associated with an improvement in functional capacity using the 6MWT and functional mobility using the TUG test. The functional capacity result in our study was comparable to a borderline significant result (*P* = 0.09) found in a recent multicentred, prematurely terminated PREHAB trial (Molenaar et al. 2023). A systematic review of three colorectal surgical RCTs (*n* = 250) of prehabilitation showed a clinically meaningful improvement in exercise capacity before surgery (25 m, 95% *CI*: 11 to 39) (Molenaar et al. 2022). Our participants’ improvement in the TUG test was clinically important when compared to the reference values estimated from a systematic review of studies in the healthy elderly population (Bohannon 2006). Nonetheless, the minimal preoperative changes in the VO_2peak_, hand grip strength, cognitive function, and emotional function results were similar to findings reported in other recent cancer prehabilitation studies (Chmelo et al. 2022; Molenaar et al. 2023 Mar). Overall, the results suggest that the multimodal 3-week prehabilitation intervention was associated with improved functional capacity using 6MWT and improved functional mobility measured by the TUG test.

There were several limitations to this pilot study. Selection bias was likely to be present as only one in three elderly participants was frail, despite using a comprehensive, inclusion criterion that included procedure-specific criteria to benefit patients, regardless of their physical fitness, undergoing high-risk major operations. As unmeasured confounding and measurement bias may be present due to the retrospective observational study design, the results should be regarded as exploratory. The target energy and protein level suggested by dietitians involved with the nutritional prehabilitation could not be guaranteed with the outpatient program. Socio-economic bias may be present as two patients, who opted to have their surgical operations done in private hospitals, were excluded from the analysis. Two dialysis patients opted out of prehabilitation due to medical disabilities and inconvenience. This may lead to an overestimation of the effect of prehabilitation programme as severely frail patients were excluded. Finally, the pilot study was not powered to detect changes in secondary outcomes even though many secondary outcomes were collected to reflect ‘frailty’ as no single accepted definition of frailty exists (Gillis et al. 2022).

The implication of the study highlights the importance of having a close, multidisciplinary collaboration between anaesthetists, geriatricians, surgeons, and allied health professionals for patients to experience a smooth perioperative journey. Good communication is essential among team members to facilitate a structured and tailored approach to optimize the physiological reserve and functional capacity of frail high-risk surgical patients over several weeks preoperatively. The study is an example service model for re-engineering perioperative service in Hong Kong. However, future alternative service models should consider revising the inclusion criteria to target more high-risk frail patients to potentially benefit from prehabilitation and to recognize travelling as a major barrier to participating in prehabilitation. We could consider an alternative inpatient prehabilitation model for patients with medical disabilities or travelling problems, such as those who require frequent dialysis or stoma care, or elderly patients with poor social networks who found frequent travelling difficult to an outpatient-based prehabilitation programme. Currently, we are completing a physical prehabilitation RCT (ChiCTR1800016098) (Yau et al. 2019), beginning a nutritional prehabilitation RCT for malnourished patients (ChiCTR2200057463), and will shortly recruit patients to a psychological prehabilitation RCT (ChiCTR2100053637) (Wong et al. 2022). These high-quality, evidence-based, initiatives will provide more understanding about the effect of prehabilitation on patient care experience and the quality of recovery after surgery.

## Conclusions

This pilot outpatient-based, one-stop multidisciplinary prehabilitation programme for high-risk surgical patients was feasible, safe, and acceptable. The multimodal 3-week prehabilitation programme was associated with improving several measures of the patient’s physiological reserve and functional capacity. The study served as an example service model for re-engineering the perioperative service in Hong Kong. Revision of the inclusion criteria and addressing the barriers to patient’s participation in prehabilitation programmes should be considered in alternative service models.

## Data Availability

Datasets used or analysed during the current study are available from the corresponding author on reasonable request.

## Abbreviations

DAH_30_: Days alive and at home within 30 days after surgery
POMC: Perioperative medicine clinic
PWH: Prince of Wales Hospital
MST: Malnutrition Screening Tool
CFS: Clinical Frailty Scale
6MWT: 6-Min walk test
DASI: Duke’s Activity Status Index
ASA-PS: Amercian Society of Anaesthesiologists Physical Status
GOLD: Global Initiative for Chronic Obstructive Lung Disease
GDH: Geriatric Day Hospital
VO_2peak_: Peak oxygen uptake
VO_2_R: Oxygen uptake reserve
TUG: Timed Up and Go
MFAC: Modified Functional Ambulatory Category
mRMI: Modified Rivermead Mobility Index
MFS: Morse Fall Scale
MBI: Modified Barthel Index for activity daily living
LawADL: Lawton Instrumental Activities of Daily Living
DASS-21: Depression Anxiety Stress Scale 21
BMI: Body mass index
SMM: Skeletal muscle mass
SD: Standard deviation
IQR: Interquatile range
RCT: Randomized controlled trial
